# Supplementary figures and images for: Virus–host interactions in carcinogenesis of Epstein-Barr virus-associated gastric carcinoma: Potential roles of lost ARID1A expression in its early stage
Source: PLoS One. 2021 Sep 1;16(9):e0256440. doi: 10.1371/journal.pone.0256440 (PMC8409614; doi:10.1371/journal.pone.0256440)

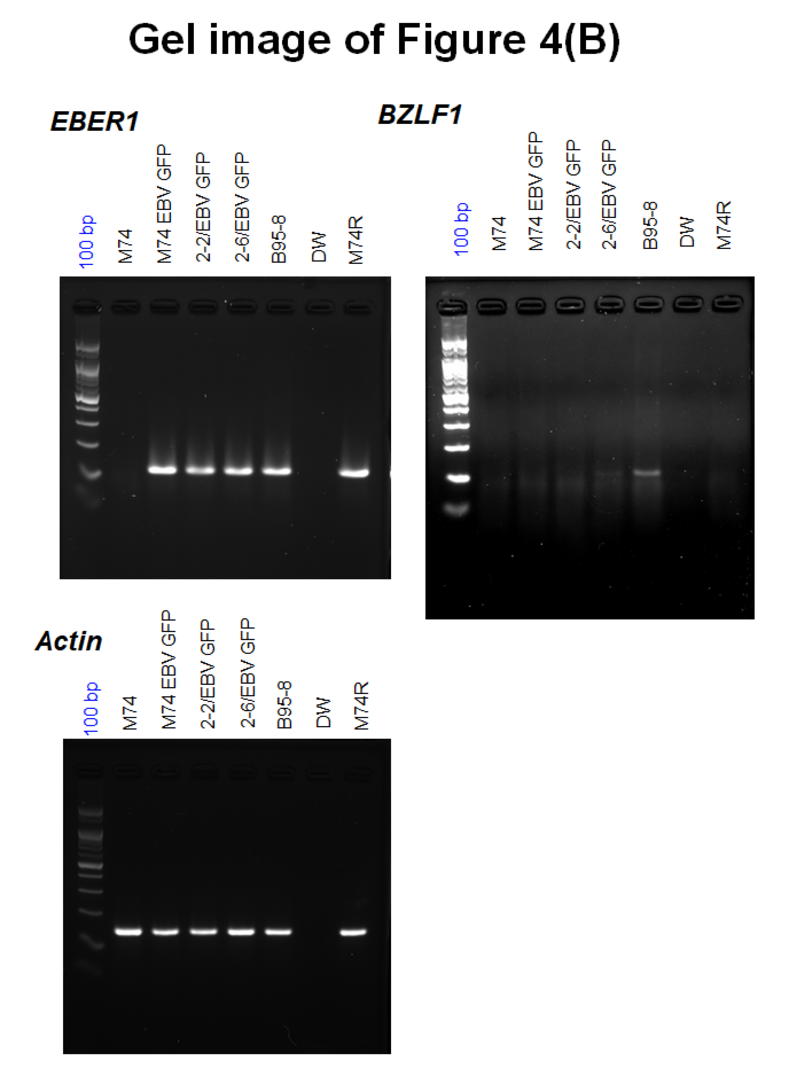

Supplement: S1 Fig — (TIF) [file pone.0256440.s001.tif]
