# Supplementary material for: Virus–host interactions in carcinogenesis of Epstein-Barr virus-associated gastric carcinoma: Potential roles of lost ARID1A expression in its early stage
Source: PLoS One. 2021 Sep 1;16(9):e0256440. doi: 10.1371/journal.pone.0256440 (PMC8409614; doi:10.1371/journal.pone.0256440)
Supplement: Attachment — Submitted filename: Response to Reviewers.doc [file pone.0256440.s002.doc]

Manuscript no.: PONE-D-21-04978

We appreciate the reviewers’ suggestions and comments. As a consequence of these valuable suggestions, we believe that our manuscript has been greatly improved. To aid in the re-review of this manuscript, we have included a point-by-point response to each of the reviewers’ comments below. Changes within the revised manuscript are underlined.

*Editor’s comments:*

*1. Other than the Reviewers’ comments, I would like to ask you to address the EBV infection status, i.e, lytic and latency, in those ARID1A modulated specimens and EBV-infected cells in vitro.*

We appreciate the Editor’s comments. We have examined the expression of BZLF1 in MKN74 cells (We also previously demonstrated absence of BZLF1 expression in EBV-infected MKN7 and MKN74 cells [Microorganisms 2020, 8, 1776]). We confirmed the latency again, and the results have been added as Figure 4(B). In addition, following sentences have been added to the manuscript:

(Page 8, line 20)

**Materials and Methods**

To examine the latency status of EBV infected cells, expression of *BZLF1* was evaluated with RT-PCR and agarose gel electrophoresis. Primers used were as follows: *BZLF1* forward, CTGGTGTCCGGGGGATAAT; *BZLF1* reverse, TCCGCAGGTGGCTGCT; *EBER1* forward, AGGACCTACGCTGCCCTAGA; *EBER1* reverse, GGGAAGACAACCACAGACAC; and *ACTB* forward, AGAAGGAGATCACTGCCCTGGCACC; *ACTB* reverse, CCTGCTTGCTGATCCACATCTGCTG.

(Page 11, Line 18)

**Results**

In both EBV-infected MKN74 parental cells and *ARID1A*-knockout MKN74 clones, expression of *BZLF1* was not observed, which means the infection status was latent (Figure 4B).

(Page 19, Line 6)

**Figure 4 legend**

(B) RT-PCR of BZLF1 gene of EBV, showing lack of expression in both EBV-infected parental MKN74 cells and ARID1A-knockout clones.

*2. Also, why you used two different methods for EBV infection?*

We appreciate the Editor’s comments. We used the classical cell-to-cell contact method for evaluation of EBV-infected gastric epithelial cells for these ten years. It uses Akata cells (derived from Burkitt lymphoma), which produce *Neo* resistant gene (Neo^r^)-inserted EBV by immunoglobulin stimuli. The other, which is applied to ARID1A-KO cells, is the method using the supernatant of AGC cells-harboring the EBV, in which EGFP gene is further inserted (GFP/ Neo^r^ EBV) (Kanda et al. J Virol 2004, 78:7004). By use of GFP fluorescence system, it is easy to assess the infection efficiency with flow cytometry without G418 selection. To clarify the purpose of using GFP/ Neo^r^ EBV, we rewrote the sentence in **Material and Method** with reference to Kanda et al. as follows.

(Page 7, Line 27)

In this experiment, EGFP-EBV infection system [17] was applied to the two *ARID1A*-knockout MKN74 clones (#1 and #2) and the parental MKN74 cell line for evaluation of infection efficiency without G418 selection [18].

In addition, we modified the sentence in Discussion.

(Page 13, Line 12)

Alternatively, with G418 selection process, the establishment of infection might be ~~have been~~ unsuccessful in a certain proportion of EGFP-positive cells in the latter. In fact, EBV could escape from infected epithelial cells during cell culture [22].

***To the comments of Reviewer #1:***

*1. Is G418-resistant colony composed of 100% EBNA-positive cells? Could you confirm EBV infection in each cell like Figure 4B?*

We appreciate the reviewer’s comments. Because we could not use reliable EBNA1 antibodies, we have performed EGFP-EBV infection to MKN74 and NUGC3 cells followed by incubation with G418 at the same concentration. We confirmed that almost all the G418-resistant cells showed green fluorescence, as shown below.

MKN74 (Bright field and GFP)

NUGC3 (Bright field and GFP)

*2. As shown in Figure 1 E and F, I agree that the expression of EBER and ARID1A is not coincident. The incidence is quite low. Is it a significant finding in EBVaGC?*

As the reviewer pointed out, it is very rare to identify small lesions showing coincidence of EBER expression and loss of ARID1A. However, we described its significance in Discussion as clearly as we could.

(Page 12, Line 3)

**Discussion**

As a reference for frequencies of precursor lesions in non-neoplastic mucosa, we could take an example of *TP53* gene abnormalities in gastric carcinogenesis, although mutation of *TP53* is an early event in gastric carcinogenesis of the chromosomal instability subtype of gastric cancer (nearly 50% of total gastric cancer) [2, 20]. During the search for p53-positive cells in non-neoplastic mucosa tissues of the stomach, Ochiai et al. identified 19 foci in 756 tissue sections (2.5%) [21]. Since EBVaGC comprises 3.5-10% of gastric cancer with one thirds showing loss of ARID1A, the relative incidence of p53-positivity and EBV infection in non-neoplastic mucosa (25:2) appeared to correspond with that in gastric cancer, suggesting that both are precursors of each subtype of gastric cancer.

*3. Are their any morphologic changes in ARID1A-KO cells compared to the parent cells?*

As shown in Figure 4, ARID1A-KO cells have fibroblast-like morphology compared to the parental cells. The morphological change is also described in the Results section.

(Page 11, Line 16)

*ARID1A* knockout induced a fibroblastic morphology with filopodia formation of various degrees (Figure 4A).

*4. “BVaGC” means EBVaGC in Table 2?*

We apologize for the typo. We have corrected the spell to “EBVaGC”.

*5. Have you examined alterations in the ARID1A gene?*

We could not perform analyses of *ARID1A* gene alterations from FFPE sections due to large size of the gene (2285 amino acids=6855bps) without hot spots of *ARID1A* mutation. Instead of gene analyses, we used immunohistochemistry, which is a well-worked surrogate marker for *ARID1A* gene mutation in gastric and ovarian carcinomas [Reference 2 and 4].

***To the comments of Reviewer #2*:**

Table2

#1:

*Q: Case3; ARID1A-preserved is mentioned to EBVaGC (SM, ARID1A-lost). What does this mean?*

Table 2 is the summary of 11 intramucosal EBVaGC. In the “Multiplicity” column, characteristics of the other carcinoma was described in double carcinoma cases. Case 3 has two carcinomas, one of which is an ARID1A-preserved intramucosal EBVaGC. The other cancer was EBVaGC with submucosal invasion, in which expression of ARID1A was lost. We added “the other carcinoma” below the “multiplicity”. We also have added the explanation to the footnotes of Table 2 as follows:

*When there are two gastric carcinomas in the same case, the characteristics of the other carcinoma was described in the “Multiplicity” column.

*Q: Case7; Are BVaGC typo?*

We apologize for the typo. We have corrected the spell to “EBVaGC”.

*Abstract*

*#2: “Screening of EBER by in situ hybridization revealed a frequency of approximately 0.2% EBER-positive epithelial cells in non-neoplastic gastric mucosa tissue samples. Six small foci of EBV-infected epithelial cells showed two types of histology: degenerated (n=3) and metaplastic (n=3) epithelial cells. One of the degenerated EBER-positive foci was located within the ARID1A-lost glands. ARID1A-lost EBVaGC (n=5) showed a tubular structure with distinct luminal formation compared with ARID1A-preserved cancer (n=7) in the proper mucosal layer, suggesting morphological continuity of both EBER-positive/ARID1A-lost epithelial cells.”*

*Q: It is hard to understand the significance of this statement for general readers. I can’t follow the relationship of morphological continuity with EBER/ARID1A status.*

We appreciate for the reviewer’s comment. We described that there are morphological differences between ARID1A-lost lesions and ARID1A-preserved lesions in both early small lesions and intramucosal carcinomas. We revised and simplified the description in Abstract and Discussion.

(Page 3, Line 10)

**Abstract**

Six small foci of EBV-infected epithelial cells showed two types of histology: degenerated (n=3) and metaplastic (n=3) epithelial cells. ARID1A was lost in the former type. In intramucosal EBVaGC, there were ARID1A-lost (n=5) and -preserved tumors (n=7), suggesting that ARID1A-lost carcinomas are derived from ARID1A-lost precursor cells in the non-neoplastic mucosa.

(Page 12, Line 19)

**Discussion:**

In our previous study, lost ARID1A was correlated with no clinicopathological factors or with adverse patient prognosis [11]. However, in the present study of the intramucosal stage of EBVaGC, there were morphological differences between ARID1A-lost and -preserved carcinomas. There was a tendency that the former showed distinct glands, while the latter showed a cord-like structure. These facts suggest that EBER-positive glands with lost and preserved ARID1A expression in the non-neoplastic mucosa are direct precursor of ARID1A-lost and ARID1A-preserved carcinomas of EBVaGC, respectively.

*Materials and Methods*

*#3: Tissue Samples:*

*Q: How did the authors select three series of samples for EBERs-screening? Also, they mention the detail of first and second series. However, they did not describe the detail for the third series. Why?*

We appreciate the reviewer’s comment. We used two consecutive cases to avoid the selection bias in first and second series (2005 to 2007 in the first series and 2010 to 2012 in the second series). The third series was a case file collected from the personal experience of one of the authors (T.U.). More specifically, EBER-ISH was performed in approximately 300 cases of surgically or endoscopically resected gastric cancer which was suspicious for EBVaGC from 1990 to 2020. In addition, consecutive 185 cases of endoscopically resected gastric cancer in 2006 were screened by EBER-ISH. We have revised the description as follows:

(Page 5, Line 19)

**Materials and Methods**

The third source was derived from the file of one of the authors (TU), in which EBER-positive cells were identified in the non-neoplastic mucosa near the gastric cancer during the screening of EBV infection. More specifically, EBER-ISH was performed in approximately 300 cases of surgically or endoscopically resected gastric cancer from 1990 to 2020 which were suspicious for EBVaGC based on the morphological characteristics. In addition, 185 consecutive cases of endoscopically resected gastric cancer were screened by EBER-ISH.

*Results*

*#4: ARID1A expression was evaluated in serial sections of these six foci of EBER-positive lesions. Two foci could not be evaluated because the EBER-positive foci disappeared during the preparation of serial sections. Three foci showed preserved ARID1A expression, and one showed lost ARID1A.*

*Q: According to the authors’ statement, ARID1A expression precede before EBV-infection. However, 3 of 4 EBER-positive cases retained ARID1A expression. Please explain.*

In EBVaGC, mutation of *ARID1A* gene and loss of ARID1A expression are observed approximately half of EBVaGC [Reference 2], and the other half of EBVaGC has preserved expression of ARID1A. We think the three EBER-positive foci with preserved ARID1A expression are the very early lesion of ARID1A-preserved EBVaGC. We have added the description as follows:

(Page 12, Line 23)

**Discussion**

These facts suggest that EBER-positive glands with lost and preserved ARID1A expression in the non-neoplastic mucosa are direct precursor of ARID1A-lost and ARID1A-preserved carcinomas of EBVaGC, respectively.

*#5: “ARID1A-lost EBVaGC showed a tubular formation more distinctly, and ARID1A-preserved EBVaGC predominantly showed a cord-like structure. Lace pattern, which is an interconnecting cord-like or tubular structure [19], was observed in both ARID1A-lost and -preserved EBVaGC”*

*Q: It is hard to understand the relevance of tissue structure and ARID1A, EBERs in EBVaGC. Please explain plainly.*

We appreciate the reviewer’s comment. We described here that EBVaGC with loss of ARID1A and preserved ARID1A have different morphological features. We have deleted the confusing sentence and revised the description in the Results and Discussion section as plainly as we could.

(Page 10, Line 21)

**Results**

ARID1A-lost EBVaGC showed a tubular formation more distinctly, and ARID1A-preserved EBVaGC predominantly showed a cord-like structure. ~~Lace pattern, which is an interconnecting cord-like or tubular structure [19], was observed in both ARID1A-lost and -preserved EBVaGC.~~

(Page 12, Line 19)

**Discussion**

In our previous study, lost ARID1A was correlated with no clinicopathological factors or with adverse patient prognosis [11]. However, in the present study of the intramucosal stage of EBVaGC, there were morphological differences between ARID1A-lost and -preserved carcinomas. There was a tendency that the former showed distinct glands, while the latter showed a cord-like structure. These facts suggest that EBER-positive glands with lost and preserved ARID1A expression in the non-neoplastic mucosa are direct precursor of ARID1A-lost and ARID1A-preserved carcinomas of EBVaGC, respectively.

*Discussion*

*#6:*

*Q: Relationship of TP53, ARID1A, and EBVaGC is not clear for me. Also, morphological relevance of ARID1A and EBER-positive cell in terms with EBV-associated carcinogenesis is not clearly described. According to the authors’ statement, ARID1A mutation, which precede to EBV infection, should be more frequent than TP53 mutation. Their statement is quite confusing to me. Please simplify the story.*

We appreciate the reviewer’s comment. Mutation of *TP53* is an early stage of event in carcinogenesis of gastric cancers with chromosomal instability (CIN), which is one of the molecular subtypes of gastric cancer other than EBVaGC. In addition, it is known that mutation of TP53 is not observed in EBVaGC. CIN was much more frequent than EBVaGC, and the ratio of small foci (TP53/EBER, 25/2) is comparable to that of CIN/EBVaGC. We have revised and simplified the description in Discussion section as follows:

(Page 11, line 26)

**Discussion**

Complex interaction between the virus and host is the primary issue in virus-associated neoplasms from the initial to the advanced stages of cancer development, which we termed the “gastritis infection carcinoma sequence of EBVaGC” [13]. In the present study, we confirmed the co-existence of EBV infection and lost ARID1A in non-neoplastic epithelial cells of the stomach. In the second series of screening, the frequency of EBER-positive glands was 0.2% in non-neoplastic mucosa tissues. As a reference for frequencies of precursor lesions in non-neoplastic mucosa, we could take an example of *TP53* gene abnormalities in gastric carcinogenesis, although mutation of *TP53* is an early event in gastric carcinogenesis of the chromosomal instability subtype of gastric cancer (nearly 50% of total gastric cancer) [2, 20]. During the search for p53-positive cells in non-neoplastic mucosa tissues of the stomach, Ochiai et al. identified 19 foci in 756 tissue sections (2.5%) [21]. Since EBVaGC comprises 3.5-10% of gastric cancer with one thirds showing loss of ARID1A, the relative incidence of p53-positivity and EBV infection in non-neoplastic mucosa (25:2) appeared to correspond with that in gastric cancer, suggesting that both are precursors of each subtype of gastric cancer.

*#7:*

*Q: They show ARID1A-lost gastric cancer cell lines gained infection efficiency. I think the data itself is interesting. However, EBV genome is frequently lost, especially in the EBV-infected epithelial cells. So it is suggested that some factors that keep EBV in the infected cell is important for the persistent infection. So, EBV might have escaped from certain proportion of gastric epithelial cells. Do you have any comments?*

We appreciate the reviewer’s suggestion. We agree with the reviewer that loss of ARID1A might activate some factors that keep EBV in the infected cells, which increased infection efficiency. Although the exact mechanism in which loss of ARID1A increased infection efficiency is hard to disclose with our data, we have added some comments in the Discussion as follows:

(Page 13, Line 13)

**Discussion**

In fact, EBV could escape from infected epithelial cells during cell culture [22]. Along the line of thinking, loss of ARID1A might contribute to keep EBV in infected cells through unknown mechanisms. Tsai et al. recently demonstrated that ARID1A loss causes decrease of recruitment of genome stability regulators such as ATR and TOP2 [23]. Further studies are also necessary to clarify interaction of EBV and chromosomal modulators [15]. Nevertheless, the results of EBV infection of *ARID1A*-knockdown and *ARID1A*-knockout cells are consistent with the comparative observation of EBER *in situ* hybridization and ARID1A immunohistochemistry.

*Other corrections*

In the original manuscript, the concentration of G418 is described as 500 µg/mL for MKN74 and 300 µg/mL for NUGC3, but the concentrations were wrong. We apologize for the mistake and have corrected the value as 300 µg/mL for MKN74 and 200 µg/mL for NUGC3 in **Materials and Methods** section.

To revise the manuscript, we have deleted two references [originally numbered as 19 and 21] and added four references [17, 20, 22, 23] to the manuscript.

17. Kanda T, Yajima M, Ahsan N, Tanaka M, Takada K. Production of high-titer Epstein-Barr virus recombinants derived from Akata cells by using a bacterial artificial chromosome system. J Virol 2004; 78:7004-7015.

20. Rokutan H, Abe H, Nakamura H, Ushiku T, Arakawa E, Hosoda F, Yachida S, Tsuji Y, Fujishiro M, Koike K, Totoki Y, Fukayama M, Shibata T. Initial and crucial genetic events in intestinal-type gastric intramucosal neoplasia. J Pathol. 2019; 247: 494-504.

22. Queen KJ, Shi M, Zhang F, Cvek U, Scott RS. Epstein-Barr virus-induced epigenetic alterations following transient infection. Int J Cancer. 2013; 132: 2076-86.

23. Tsai S, Fournier L-A, Chang EY, Wells JP, Minaker SW, Zhu YD, Wang AY, Wang Y, Huntsman DG, Stiring PC. ARID1A reulates R-loop associated DNA replication stress. PLos Genet. 2021; 17:e1009238.

In **Acknowledgements** section, we have added Keisuke Matsusaka (Department of Pathology, Chiba University) for his excellent advice.

(Page 14, Line 2)

**Acknowledgments**

The authors thank Keisuke Matsusaka (Department of Pathology, Chiba University) for his excellent advice, and Kei Sakuma, Minato Murata, Kimiko Takeshita, and Tomoko Irisa for their excellent technical assistance.
